# Supplementary material for: Health systems performance in health outcomes, health financing and COVID-19 pandemic: Lessons from 31 countries
Source: PLoS One. 2025 Oct 30;20(10):e0334693. doi: 10.1371/journal.pone.0334693 (PMC12574844; doi:10.1371/journal.pone.0334693)
Supplement: Attachment — Submitted filename: lessons from 31 countries.docx [file pone.0334693.s002.docx]

**Title: Health Systems Performance in Health Outcomes, Health Financing and COVID-19 Pandemic: Lessons from 31 Countries**

This manuscript evaluates and ranks the health system performance (HSP) of 31 countries across multiple dimensions—health outcomes, financial indicators, and COVID-19-related metrics—using the TOPSIS method and hierarchical clustering. The study highlights substantial variation in performance, with Luxembourg, Switzerland, and Qatar ranking highest, while several low-income or politically unstable countries rank lowest.

The topic is both important and timely, addressing global health system performance, especially in the context of the COVID-19 pandemic. The combination of TOPSIS and entropy weighting, along with hierarchical clustering, offers a structured, quantitative approach that is of interest to researchers and policymakers. The multi-dimensional framework—integrating health, financial, and pandemic indicators—adds to its comprehensiveness.

**Justification for Country Selection**
The selection criteria for the 31 countries need a clearer explanation. Although the authors mention purposive sampling, they should provide transparent inclusion/exclusion criteria, especially regarding the omission of some OECD or LMIC countries.

**Weighting Approach**
The use of a combined Three-Scale and Entropy-Weighting method is innovative but requires further clarification. How were the weights validated or benchmarked? The manuscript should specify if Ci (similarity index) ranges between 0 and 1 and clarify the scale in Table 2 to ensure it is self-explanatory.

**Comparability of Countries**
The manuscript aims to compare Iran’s health system with others. For meaningful comparisons, countries with similar settings (e.g., population size, health system structure, economy) should be selected. Comparing countries like Norway and Sweden is appropriate due to similar systems, but comparing Norway with Uganda is less informative due to fundamentally different contexts. Given that Iran's Ci score is close to countries like Syria, Paraguay, Kenya, and Nigeria, comparisons should focus on such peers. There will be more information when compared to similar countries like Iran, rather than high CI score countries, which have a very different system from the Iranian health care system.

**Performance Categorization**
Based on Table 2, Luxembourg, Switzerland, and Qatar clearly form the high-performance group. The middle-performance group could reasonably extend at least up to Bahrain, while the remaining countries would fall into a low-performance category. The current clustering requires reconsideration or stronger justification. It does not seem appropriate to categorize countries such as Singapore, the UK, Canada, and Australia as low performers alongside countries like Afghanistan, Nigeria, and Uganda, given the substantial differences in their health system structures, resources, and outcomes.

I recommend either revising the clustering approach or introducing an additional category (e.g., creating four performance levels instead of three) to more accurately reflect these differences. Table 2 shows that countries from the USA to Bahrain have relatively higher Ci scores, while countries from Chile to Yemen have scores that are very close to zero, which could justify labeling them as “very low performers” or “lowest performers.”

Additionally, there appears to be an inconsistency between Section 4.2 (page 16), where these countries are described as high performers, and Table 1, where they are categorized as low-performance countries. This discrepancy should be addressed to ensure clarity and alignment between the analysis and interpretation.

**Use of Data Years**
Table 3 uses data from 2000 and 2019 for MMR and NMR. The authors should explain why more recent data (if available) weren’t used to enhance the study’s relevance.

Minor Suggestions

Improve clarity and grammar throughout the manuscript.

Enhance figures/tables with clearer legends, axis labels, and explanations.

The manuscript provides valuable insights into global health system performance using a rigorous methodological approach. However, addressing the above recommendations would strengthen its clarity, methodological transparency, and policy relevance.
